# Hospitalizations among World Trade Center Health Registry Enrollees Who Were under 18 Years of Age on 9/11, 2001–2016

**DOI:** 10.3390/ijerph18147527

**Published:** 2021-07-15

**Authors:** Lisa M. Gargano, Sean H. Locke, Howard E. Alper, Jennifer Brite

**Affiliations:** World Trade Center Health Registry, Division of Epidemiology, New York City Department of Health and Mental Hygiene, Long Island City, NY 11101, USA; lisa.gargano@gmail.com (L.M.G.); halper@health.nyc.gov (H.E.A.); jbrite@health.nyc.gov (J.B.)

**Keywords:** 9/11 disaster, physical and mental health, PTSD, adolescent health

## Abstract

Much of the literature on hospitalizations post-September 11, 2001 (9/11) focuses on adults but little is known about post-9/11 hospitalizations among children. Data for World Trade Center Health Registry enrollees who were under 18-years old on 9/11 were linked to New York State hospitalization data to identify hospitalizations from enrollment (2003–2004) to December 31, 2016. Logistic regression was used to analyze factors associated with hospitalization. Of the 3151 enrollees under age 18 on 9/11, 243 (7.7%) had at least one 9/11-related physical health hospitalization and 279 (8.9%) had at least one 9/11-related mental health hospitalization. Individuals of non-White race, those living in New York City Housing Authority housing, those exposed to the dust cloud on 9/11, and those with probable 9/11-related PTSD symptoms were more likely to be hospitalized for a 9/11-related physical health condition. Older age and having probable 9/11-related PTSD symptoms at baseline were associated with being hospitalized for a 9/11-related mental health condition. Dust cloud exposure on 9/11 and PTSD symptoms were associated with hospitalizations among those exposed to 9/11 as children. Racial minorities and children living in public housing were at greater risk of hospitalization. Continued monitoring of this population and understanding the interplay of socioeconomic factors and disaster exposure will be important to understanding the long-term effects of 9/11.

## 1. Introduction

The September 11, 2001 (9/11) terrorist attacks on the World Trade Center (WTC) in New York City (NYC) and in the months that followed exposed individuals living or working in the area to both a complex mixture of dust, debris, and jet fuel combustion byproducts [1] as well as traumatic exposures, such as seeing the planes hitting the buildings, witnessing people jump from the buildings, and the death of a loved one [2]. This exposed population included over 25,000 children who lived and/or attended school in downtown Manhattan [1], a large number of whom continued to reside near the WTC site in the months after the tragedy. 

Previous research on children exposed to 9/11 found a range of physical and mental health effects. Several studies found new or worsening respiratory symptoms [2,3] as well as post-9/11 asthma diagnosis [2,3,4], both of which were associated with exposure to the dust cloud [2,3,4]. Among the Registry enrollees under the age of five, 5.7% reported new asthma diagnoses after 9/11. While age-specific asthma prevalence before 9/11 in enrolled children was similar to national estimates, the asthma prevalence was elevated among enrollees at the time of interview [2]. Other studies which focused on mental health have found a range of outcomes associated with 9/11-exposure. A study of NYC high school students six months after the WTC attack found increased drinking was associated with direct exposure to the attack [5]. Chemtob et al. (2009) found that adolescents with one WTC exposure risk factor had a five-fold increase in substance use, while those with three or more exposure risk factors had a nearly 19-fold increase [6]. Another study found adolescents who witnessed a disturbing event on 9/11 were twice as likely to report ever drinking and almost three times as likely to have ever used marijuana [7]. Among those >5 years of age on 9/11, fear for personal safety on 9/11 was significantly associated with having ever smoked cigarettes, ever drank, and ever used marijuana [7]. Exposure to 9/11, including family exposure, has also been shown to be associated with mental health conditions, such as post-traumatic stress disorder (PTSD) symptoms, depression, anxiety, and behavior problems [8,9,10]. PTSD has been shown to be associated with subsequent physical and mental health: Incidence of stroke was found to be higher among those with PTSD [11]. A study utilizing impulse oscillometry to measure lung function concluded that PTSD may contribute to lower respiratory symptoms persistence [12]. Also, approximately one third of rescue/recovery workers with PTSD have cognitive impairment regardless of 9/11 exposure level [13]. Furthermore, adolescent PTSD symptoms, for example, was significantly associated with WTC exposure 6–7 years after 9/11 [9]. PTSD symptoms and behavior problems have been associated with poor school-functioning among 9/11-exposed adolescents [14]. 

The few studies that used objective measures among 9/11-exposed children focused on clinical tests. Szema et al. (2009) investigated asthma diagnosis, medication use, and limited spirometry data in a group of schoolchildren in NYC’s Chinatown, and reported increased asthma medication use with greater proximity to the WTC site [15]. A study of children an average of 7.8 years after 9/11 reported new onset provider-diagnosed asthma in 21.4% of children, and found that dust cloud exposure was associated with pulmonary function abnormalities, such as isolated low forced vital capacity pattern and an obstructive pattern consistent with asthma [16]. Another study by Trasande et al. (2017) found an increase in serum perfluoroalkyl substances (PFASs), which play a key role in lipid and carbohydrate metabolism, in 9/11-exposed children compared to matched controls and that this increase was associated with dust exposure at home and traumatic experiences [17]. A subsequent analysis showed that these PFASs were positively associated with triglycerides, total cholesterol, LDL cholesterol, and decrease insulin resistance, all early markers for atherosclerosis and cardiovascular diseases [18].

There are no published studies on hospitalizations, to our knowledge, among individuals who were exposed to 9/11 as children. This study sought to (1) describe patterns of hospitalization among enrollees in the World Trade Center Health Registry (WTCHR) who were under 18 years of age on 9/11 and (2) to assess whether 9/11-related dust exposure or PTSD symptoms are associated with increased odds of hospitalization. Given that previous research has shown that exposure to 9/11 is associated with physical health outcomes, such as asthma diagnosis, and mental health outcomes, such as PTSD and substance use, we hypothesize that hospitalization for these conditions is also associated with 9/11-exposure.

## 2. Materials and Methods

### 2.1. Study Population

The study population was drawn from the World Trade Center Health Registry (WTCHR), a cohort study of over 71,000 individuals exposed to the September 11th World Trade Center attacks in New York City. In the present longitudinal study, the WTCHR includes rescue and recovery workers; lower Manhattan residents living south of Canal Street; school children, building occupants, or passersby south of Chambers Street [19]. There were 3151 enrollees who were under the age of 18 on 9/11. This constitutes the analytic sample for the present study. Data collection for the baseline survey was from 2003–2004. Enrollees who were still under 18 years of age at the time of the baseline survey had responses provided by a parent or guardian. Those 18 years or older at baseline completed the survey themselves [2]. The survey included questions on demographic information, 9/11-exposure, as well as physical and mental health status.

### 2.2. Statewide Planning and Research Cooperation System

Hospitalizations among enrollees were identified through a linkage to the New York State Department of Health’s Statewide Planning and Research Cooperation System (SPACRS) database. The SPARCS program is an administrative reporting system that includes approximately 95% of hospital discharges in New York since 1991 [20]. The WTCHR enrollees were linked to the available SPARCS data. Hospital discharge data were available between 11 September 2001 and 31 December 2016 and emergency department (ED) visits were available between 1 January 2005 and 31 December 2016. Records were matched based on an algorithm which used parts of the name and Social Security number and full date of birth, gender, and zip code.

### 2.3. Hospitalization Outcomes

This study evaluated hospitalizations for those enrollees who were under 18 years of age on 9/11 for both physical and mental health conditions reported in admitting, principal, and other diagnosis. Enrollees with multiple physical health conditional related hospitalization were treated as having a single such hospitalization. A similar approach was used for mental health condition hospitalizations. ICD-9 codes were employed to indicate disease status. ICD-10 codes were only available for one year of the matching and thus were not used. For 9/11-related physical health hospitalizations, conditions that had previously been assessed as associated with 9/11-exposure in adults by either self-report or hospitalization records were examined [21,22,23,24,25,26]. The conditions were asthma (ICD-9 code 493.xx) as the discharge diagnosis or as a hospitalization with another respiratory condition listed as the principal discharge diagnosis [23], gastro-esophageal reflux disease (GERDS) (ICD-9 code 530.81, 530.10, 530.11, 530.19, and 530.3) [25,26,27], and cardiovascular diseases (ICD-9 codes, 401–405, 410–414, 427, 428, 430–438) [22]. Due to the small number of enrollees with hospitalizations for these conditions (asthma, *n* = 172; GERDS, *n* = 38; cardiovascular diseases, *n* = 53), they were combined into one variable indicating ≥1 hospitalization for 9/11-related physical health condition variable. Participants with multiple 9/11-related physical health hospitalizations would only be counted once in the study sample.

For 9/11-related mental health conditions, hospitalizations for mental disorders (ICD-9 codes 290–319), including drugs and alcohol (265.2, 303, 304, 357.5, 425.5, 535.3, 305.0, 305.2–305.9, 965, 967, 969, 970, 968.0, 968.5, 980.0, 291, 292, and 571.0–571.9, except for 571.5) were combined into one variable. Participants with multiple 9/11-related mental health hospitalizations would only be counted once in the study sample. 

### 2.4. 9/11 Exposures

All exposure information was collected on the baseline survey. Dust cloud exposure was defined as having been outdoors within the dust or debris cloud that resulted from the collapse of the WTC towers in baseline survey (2003–2004). Probable post-traumatic stress disorder (PTSD) like symptoms was defined using different sets of questions depending on age at baseline. For those enrollees under 18 years of age at baseline PTSD symptoms was defined as parent report of the child having at least six of eight 9/11-specific stress symptoms. For enrollees who were under 18 on 9/11 but 18 years or older at the time of baseline, PTSD symptoms was defined as a score or 44 or greater on a 9/11-specfic PCL-17 [28]. Dust cloud exposure and PTSD symptoms were chosen as independent variables based on consistency with prior 9/11 literature and previous associations found among adult populations.

### 2.5. Sociodemographic Variables

All demographic covariates were measured at baseline and included age on 9/11, gender, and race/ethnicity. Though the latter variable consisted of White, Black, Hispanic, Asian, and other race/ethnicity enrollees, we further categorized race/ethnicity as white/non-white. Due to the small sample size for this study, non-white racial/ethnic groups had small sample sizes precluding statistical analyses of the association of specific racial/ethnic groups with hospitalization. As a proxy measure for socio-economic status, address data collected was geocoded and an indicator was created for whether an enrollee lived in NYC Housing Authority (NYCHA) housing. NYCHA provides housing for low- and moderate-income residents throughout the five boroughs of NYC. NYCHA also administers a citywide Section 8 Leased Housing Program in rental apartments.

### 2.6. Statistical Analysis

Among those with 9/11-related physical or mental health hospitalization, separate multivariable logistic regressions were used to calculate adjusted odds ratios for the associations between sociodemographic variables, 9/11-related PTSD symptoms, dust cloud exposure, and being hospitalized with a 9/11-related physical or mental health hospitalization. The ‘no” category included those who had a hospitalization, just not for the indicated group of 9/11-related conditions, and enrollees with no hospitalizations at all. Analyses were conducted using SAS 9.4 (SAS Institute, Cary, NC, USA). Statistical significance was set at a two-sided *p*-value of less than 0.05.

## 3. Results

### 3.1. Study Population Characteristics 

There were 3151 enrollees in the analytic sample, of which 1705 had at least one hospitalization during the study period, for a total of 6945 hospitalizations (Table 1). The largest proportions were among female (50.5%) and non-White groups (including Black, Hispanic Asian, and other non-White race/ethnic groups not specified) (53.2%), and those who did not live in NYCHA housing (85.4%) (Table 1). The mean age on 9/11 was 9.1 years. Regarding 9/11-exposures, almost half were immersed in the dust cloud on 9/11 and 3.5% had probable 9/11-related PTSD symptoms. 

Of the total enrollees hospitalized, 243 (7.7%) had at least one 9/11-related physical health hospitalization, totaling 458 hospitalizations (Table 2). Of those hospitalized for a 9/11-related physical health condition, the largest proportion were among male, Black, Hispanic, Asian, other race, and non-NYCHA housed enrollees. The mean age on 9/11 was 9.6 years. Over half of those with a 9/11-related physical health condition hospitalization were exposed to the dust cloud on 9/11 and 7.6% had probable 9/11-related PTSD symptoms. Among those who were hospitalized for a 9/11-related physical health condition, 65.4% had one hospitalization, 16.5% had two, and 18.1% had three or more.

There were 279 (8.9%) enrollees with at least one 9/11-related mental health hospitalization, totaling 538 hospitalizations. Similar to 9/11-related physical health hospitalization, the largest proportion were among male, non-White, and non-NYCHA housed enrollees. The mean age on 9/11 was slightly higher at 10.4 years. Less than half were exposed to the dust cloud on 9/11 and 9.3% had probable 9/11-related PTSD symptoms (Table 2). For 9/11-related mental health hospitalization, 64.2% had one, 16.9% had two, and 19.0% had three or more.

### 3.2. Factors Associated with Physical and Mental Health Condition Hospitalization

Compared to White enrollees, non-White enrollees were 55% more likely to be hospitalized for a 9/11-related physical health condition (adjusted odds ratio (AOR): 1.55, 95% confidence interval (CI): 1.06–2.28) (Table 3). Enrollees who lived in NYCHA housing at baseline were more than three times as likely to be hospitalized for a 9/11-related physical health condition (AOR: 3.16, 95% CI: 2.18–4.58) compared to those who did not live in NYCHA housing. Those who were exposed to the dust cloud on 9/11 were 54% more likely to have at least one 9/11-related physical health condition hospitalization (AOR: 1.54, 95% CI: 1.12–2.12). Probable 9/11-related PTSD symptoms were also significantly associated with 9/11-related physical health hospitalization (AOR: 1.88, 95% CI: 1.02–3.44).

Older age on 9/11 was associated with having at least one hospitalization for a 9/11-related mental health condition, with each additional year resulting in 6% increased odds (AOR: 1.05, 95% CI: 1.02–1.08) (Table 3). Being in NYCHA housing, race/ethnicity, and dust cloud exposure on 9/11 were not associated with 9/11-related mental health hospitalization. Enrollees who had probable 9/11-related PTSD symptoms at baseline were three times more likely to have a 9/11-related mental health hospitalization, compared to those who did not have 9/11-related PTSD symptoms (AOR: 3.01, 95% CI: 1.74–5.22). 

## 4. Discussion

Among WTCHR enrollees who were under 18 years of age on 9/11, those who lived in NYCHA housing, were exposed to the dust cloud, or had 9/11-related PTSD symptoms were more likely to be hospitalized for a 9/11-related physical health condition. Those who were non-White (including Black, Hispanic, Asian, or other non-specified race/ethnic groups) were also more likely to be hospitalized for a 9/11-related physical health condition. In addition, enrollees of older age and those with 9/11-related PTSD symptoms were more likely to be hospitalized for a 9/11-related mental health condition. This study is one of the few that used an objective measure of the health outcomes among those who were children on 9/11.

In the current study, hospitalization for a 9/11-related physical health condition consisted of several conditions: asthma, GERDS, and cardiovascular diseases. This study showed that hospitalization for a 9/11-related physical health condition was associated with exposure to the dust cloud. This finding is similar to studies conducted in adult populations exposed to 9/11. A study by Lin et al. (2010) found an increase in respiratory, cardiovascular, or cerebrovascular illness hospitalization in the weeks following 9/11 among lower Manhattan residents compared to a control group in Queens [29]. A study among adults in the WTCHR found an association between self-reported cardiovascular disease and dust cloud exposure [21]. A subsequent study found that high overall 9/11-exposure, which included dust cloud exposure, was associated with cardiovascular disease hospitalization among adult WTCHR enrollees [22]. Numerous studies among adults and children exposed to 9/11 identified a relationship between dust exposure and asthma [2,3,4,30,31]. However, a study among 9/11-exposed adults did not find an association between asthma hospitalization and dust cloud [23]. Previous research on self-reported asthma control among 9/11-exposed adults and children found that dust cloud exposure was not associated with the level of asthma control [4,32]. More long-term studies are needed to determine the impact of dust cloud exposure on asthma control and hospitalization. Several studies have found an association between self-reported GERDS and dust cloud exposure among 9/11-exposed adults [25,26]. One of the few studies on individuals who were under 18 on 9/11 was a study of patients who presented at the WTC Environmental Health Center/Survivors Health Program, which found that dust cloud exposure was associated with decreased spirometry and home dust exposure was associated with reduced high-density lipoprotein and elevated triglycerides [16]. Other studies by Trasande et al. found that, compared to a control group, WTCHR enrollees who were under 18 on 9/11 had higher levels of early markers of atherosclerosis and cardiovascular diseases [17,18]. 

We found that demographic characteristics, themselves proxies for structural racism, known to be risk factors for either self-reported disease and/or hospitalization for physical health conditions of interest, such as asthma, GERDS, cardiovascular disease, including race/ethnicity and socioeconomic status, were significantly associated with hospitalization for a 9/11-related physical health condition [33,34,35]. Our findings align with previous 9/11 research showing that both minority children and adults and those from low SES backgrounds were at higher risk of asthma [2,3,23]. In addition, a previous study found that children from lower income households were more likely to have uncontrolled asthma [4], which could lead to ED visits. A recent study found that race/ethnicity and income were associated with asthma-related ED visits among adults exposed to 9/11, independent of barriers to care [36]. Although the relationship between socioeconomic factors and health has been documented extensively [37,38], relatively little research has been conducted in disaster-exposed populations. The results presented here demonstrate that the effects of systemic racism and health disparities extend to such populations and suggest effective disaster response should take such racism-derived disparities into consideration in order to achieve health equity. Those subjected to such disparities may have greater difficulty meeting their needs following a disaster, necessitating the prioritization of these households [39]. Such subpopulations may need additional interventions and resources, such as language support, legal and financial aid, long-term housing assistance, and social services, to supplement standard disaster relief. 

We found an elevated and significant odds of physical health condition hospitalization and 9/11-related PTSD symptoms. A growing body of literature links 9/11-related PTSD symptoms to physical health illness and/or hospitalization, such as asthma, asthma control, GERDS, and cardiovascular diseases [21,26,30,31,32]. Mental health conditions have been linked to lower adherence to asthma control medications in both adults and children exposed to 9/11 [4,32]. Other self-management behaviors may also be impacted by mental health [40,41]. Serious mental illness has been found to predict subsequent hospitalization for asthma exacerbations [42]. Potential mechanisms for the relationship between PTSD symptoms and cardiovascular disease include increases in blood pressure and lipid metabolism [43,44,45], as well as behaviors such as smoking and alcohol consumption [46,47]. Targeted interventions may be helpful for 9/11-exposed populations with comorbid mental and physical health conditions to educate them on the importance of understanding how these conditions can impact each other. 

In addition to hospitalization for a physical health condition, this study also assessed hospitalizations due to mental health conditions which included mental health illnesses and drug- and alcohol-related hospitalizations. Similar to previous work, older age was associated with a mental health condition hospitalization [24]. We found that having 9/11-related PTSD symptoms as measured at baseline (2003–2004) was associated with having at least one hospitalization for a mental health condition. A study among adults in the WTCHR found that 9/11-related PTSD symptoms were associated with both drug- and alcohol-related hospitalization [24]. Our findings are in line with reports from both 9/11-exposed adults and children that 9/11-related PTSD symptoms is linked to increased odds of alcohol consumption, including binge drinking [5,6,8].

This study is subject to several limitations. First, SPARCS does not include hospitalizations outside New York state or those at federal or psychiatric hospitals, therefore we were unable to include these hospitalizations. Second, the relatively small number of enrollees with physical or mental health condition hospitalizations necessitated the grouping of hospitalizations and may have limited our ability to detect significant differences. Also, the exposure data might be subject to recall bias since the interview has been conducted three years after exposure. In addition, a comparison group is very difficult to obtain for this cohort of children, particularly more than a decade after the disaster took place. Finally, both 9/11-related PTSD symptoms and dust cloud exposure were collected by proxy report by parent or caregiver. While the use of parent proxy report is common for children who are young because of cognitive abilities [48], research on child–parent agreement has shown mixed findings, with some studies finding high child–parent agreement and some low [49,50].

Despite the limitations, this study also has several strengths. The use of SPARCS data allowed for examination of clinically validated, objective endpoints, rather than relying on self-report, making it one of the few studies on those under 18 years of age on 9/11 to do so. In addition, we benefited from a long follow-up period and no loss to follow-up because only baseline surveys were used.

These findings are applicable to the current practice of public health. The present results suggest that children exposed to trauma may experience health consequences across the life course. Future disaster response efforts should therefore consider health care needs that may arise years or even decades after initial exposure to traumatic events. From a public health perspective, this may entail conducting surveillance and providing resources to affected populations on a much longer timescale than is existing standard practice. From a clinical perspective, the current study highlights the need for practitioners to utilize guidelines for trauma informed care [51,52].

## 5. Conclusions

Up to 15 years after 9/11, we found that dust cloud exposure, having 9/11-related PTSD symptoms, and being of Black, Hispanic, Asian, or other non-specified racial/ethnic groups were associated with a significantly higher risk for hospitalization for a 9/11-related physical health condition and that 9/11-related PTSD symptoms were associated with hospitalization for a 9/11-related mental health condition. Long-term monitoring and follow-up of those who were children when exposed to 9/11 continues to be warranted with a particular focus on social factors that may exacerbate the complex relationship between trauma and health throughout the life course.

## Figures and Tables

**Table 1:** Characteristics of World Trade Center Health Registry enrollees residing in New York State who were <18 years of age on 9/11.

Variable	Total
	N (%)
Total enrollees	3151 (100)
Total hospitalizations	6945
Age on 9/11 (years)	
Mean (SD)	9.1 (5.3)
Sex	
Male	1561 (49.5)
Female	1590 (50.5)
Race/Ethnicity	
Non-White ^a^	1675 (53.2)
White, non-Hispanic	1476 (46.8)
NYCHA ^b^	
Yes	388 (14.6)
No	2265 (85.4)
Exposure to dust cloud	
Yes	1412 (45.8)
No	1672 (54.2)
9/11-related PTSD symptoms	
Yes	91 (3.5)
No	2530 (95.5)

SD—Standard deviation. **^a^** Non-white race/ethnicity includes Black, Hispanic, Asian, and other groups. ^b^ enrollees have lived in NYC Housing Authority (NYCHA) housing.

**Table 2:** Distribution of 9/11-related physical and mental health conditions vs. hospitalizations, 11 September 2001–31 Decmeber 2016.

	No (0 Hospitalizations), Yes (≥1 Hospitalizations)			
	9/11-Related Physical Health Condition		9/11-Related Mental Health Condition	
Variable	Yes	No ^b^	Yes	No ^b^
	N (%)	N (%)	N (%)	N (%)
Total enrollees ^a^	243 (7.7)	2908 (92.3)	279 (8.9)	2872 (91.1)
Total hospitalizations	458	0 (?)	538	0 (?)
Sex				
Male	138 (56.8)	1423 (48.9)	145 (52.0)	1415 (49.3)
Female	105 (43.2)	1485 (51.1)	134 (48.0)	1456 (50.7)
Race/Ethnicity				
Non-White ^c^	170 (70.0)	1505 (51.7)	171 (61.3)	1504 (52.4)
White, non-Hispanic	73 (30.0)	1403 (48.3)	108 (38.7)	1368 (47.6)
NYCHA ^d^				
Yes	79 (37.1)	309 (12.7)	52 (21.6)	336 (13.9)
No	134 (62.9)	2131 (87.3)	189 (78.4)	2076 (86.1)
Exposure to dust cloud				
Yes	126 (52.5)	1286 (45.2)	126 (45.7)	1286 (45.8)
No	114 (47.5)	1558 (55.8)	150 (54.3)	1522 (54.2)
9/11-related PTSD symptoms				
Yes	16 (7.6)	75 (3.1)	22 (9.3)	69 (2.9)
No	194 (92.4)	2336 (96.9)	214 (90.7)	2316 (97.1)
	Mean (SD)	Mean (SD)	Mean (SD)	Mean (SD)
Age on 9/11 (years)	9.6 (5.4)	9.0 (5.4)	10.4 (4.8)	8.9(5.4)

^a^ Row %, remainder are column %; **^b^** The ‘no” category includes those who had a hospitalization that was not for the indicated group of conditions (e.g., physical or mental disease as defined in the text), plus those with no hospitalizations of any kind; SD—Standard deviation; **^c^** Non-white race/ethnicity includes Black, Hispanic, Asian, and other groups. ^d^ enrollees have lived in NYC Housing Authority (NYCHA) housing.

**Table 3:** Crude (COR) and Adjusted odds ratios (AOR) for 9/11-related physical and mental health condition hospitalizations in New York State among World Trade Center Health Registry enrollees who were ≤18 years of age on 9/11, 11 September 2001–31 Decmeber 2016.

**9/11-Related Physical Health Condition**								
		**Unadjusted**				**Adjusted**		
	**β**	**COR (95% CI)**	**SE**	***p***	**β**	**AOR (95% CI)**	**SE**	***p***
Age on 9/11	0.02	1.02 (0.99–1.04)	0.01	0.14	0.01	1.01 (0.99–1.04)	0.02	0.36
Sex								
Male	0.16	1.37 (1.05–1.79)	0.07	0.02	0.08	1.17 (0.86–1.61)	0.08	0.32
Female	Reference	Reference	Reference	Reference	Reference	Reference	Reference	Reference
Race/Ethnicity								
Non-White ^a^	0.39	2.17 (1.64–2.88)	0.07	<0.0001	0.22	1.55 (1.06–2.28)	0.10	0.03
White, non-Hispanic	Reference	Reference	Reference	Reference	Reference	Reference	Reference	Reference
NYCHA ^b^								
Yes	0.70	4.07 (3.00–5.50)	0.08	<0.0001	0.57	3.16 (2.18–4.58)	0.09	<0.0001
No	Reference	Reference	Reference	Reference	Reference	Reference	Reference	Reference
Exposure to dust cloud								
Yes	0.15	1.34 (1.03–1.74)	0.07	0.03	0.22	1.54 (1.12–2.12)	0.08	0.01
No	Reference	Reference	Reference	Reference	Reference	Reference	Reference	Reference
9/11-related PTSD symptoms								
Yes	0.47	2.57 (1.47–4.50)	0.14	0.00	0.31	1.88 (1.02–3.44)	0.14	0.04
No	Reference	Reference	Reference	Reference	Reference	Reference	Reference	Reference
**9/11-Related Mental Health Condition**								
		**Unadjusted**				**Adjusted**		
	**β**	**COR (95% CI)**	**SE**	***p***	**β**	**AOR (95% CI)**	**SE**	***p***
Age on 9/11	0.05	1.05 (1.03–1.08)	0.01	<0.0001	0.05	1.05 (1.02–1.08)	0.01	0.00
Sex								
Male	0.05	1.11 (0.87–1.42)	0.06	0.40	0.02	1.05 (0.78–1.41)	0.08	0.76
Female	Reference	Reference	Reference	Reference	Reference	Reference	Reference	Reference
Race/Ethnicity								
Non-White ^a^	0.18	1.44 (1.12–1.85)	0.06	0.00	0.06	1.13 (0.81–1.58)	0.08	0.46
White, non-Hispanic	Reference	Reference	Reference	Reference	Reference	Reference	Reference	Reference
NYCHA ^b^								
Yes	0.27	1.70 (1.23–2.36)	0.08	0.00	0.20	1.49 (0.99–2.23)	0.10	0.05
No	Reference	Reference	Reference	Reference	Reference	Reference	Reference	Reference
Exposure to dust cloud								
Yes	0.00	0.99 (0.78–1.27)	0.06	0.96	−0.06	0.88 (0.65–1.19)	0.08	0.41
No	Reference	Reference	Reference	Reference	Reference	Reference	Reference	Reference
9/11-related PTSD symptom								
Yes	0.62	3.45 (2.09–5.69)	0.13	<0.0001	0.55	3.01 (1.74–5.22)	0.14	<0.0001
No	Reference	Reference	Reference	Reference	Reference	Reference	Reference	Reference

Bolded—significant at *p* < 0.05; Adjusted for all variables in the table; **^a^** Non-white race/ethnicity includes Black, Hispanic, Asian, and other groups. ^b^ enrollees have lived in NYC Housing Authority (NYCHA) housing.
